# Carneiro’s global dataset of cultural traits in ethnographic and historical societies: a digital edition

**DOI:** 10.1038/s41597-026-08212-0

**Published:** 2026-10-02

**Authors:** Václav Hrnčíř, Robert Forkel, Russell D. Gray

**Affiliations:** 1 Department of Linguistic and Cultural Evolution, Max Planck Institute for Evolutionary Anthropology, Leipzig, Germany; 2 School of Psychology, University of Auckland, Auckland, New Zealand

## Abstract

The late Robert L. Carneiro was a prominent anthropologist who devoted his life to the study of political evolution and the evolution of complex societies. In the 1960s and 1970s, he and his colleagues created a cross-cultural dataset for their Scale Analysis project, collecting ethnographic and historical data on hundreds of cultural traits in more than a hundred societies worldwide. Although Carneiro presented the results of the project in several papers, the dataset itself has not yet been published. Here we present a digital version of two editions of the dataset, based on Carneiro’s documentation stored in the archives of the American Museum of Natural History. The 4th edition contains 127 societies and 354 cultural traits, and the 6th edition features 74 societies and 618 traits. Both represent unique sets of coded cross-cultural information that can be used as a comparative source to other cross-cultural datasets, as a starting point for one’s own collection of cross-cultural data, and for quantitative testing of cultural evolutionary hypotheses.

## Background & Summary

Human cultures differ in countless ways, be it the languages they speak, the foods they eat, the dwellings they inhabit, the institutions they create, the technologies they use, the gods they worship, or the moral and social norms they follow. Describing and explaining cultural diversity across space and time has been a major objective of the social sciences, and cross-cultural datasets provide a powerful tool for this purpose^1^. Unlike field site comparisons, quantitative cross-cultural datasets can encompass a large sample of cultural groups, enabling broader and more geographically diverse comparisons. Furthermore, if they focus on ethnographically documented groups in the colonial contact era, they can provide invaluable insights into cultural diversity before the homogenizing processes of globalization. Systematically aggregating cross-cultural information and transforming it into quantitatively coded variables enables a wide range of comparative research^2^. This data can be used to explore patterns of cultural similarity and diversity, investigate trajectories of cultural change, understand the factors shaping these patterns and driving these changes, and answer many longstanding questions of cultural evolution^3,4^.

Scholars began cataloguing ethnographic data on small-scale, non-industrial societies during the 20th century. One of the first initiatives was the monographic series *Cultural Element Distributions*, which focused on North American societies and was published by the University of California in the 1930s and 1940s^5^. This was followed by the activities of George Murdock and his colleagues at the Human Relations Area Files^6^, who created both a database of subject-indexed^7^ ethnographic texts *HRAF Collection of Ethnography* and several global cross-cultural datasets, including the *Ethnographic Atlas*^8^ and the *Standard Cross-Cultural Sample*^9^. Also worth mentioning are Robert Textor’s *A Cross-Cultural Summary*^10^, Joseph Jorgensen’s Western North American Indian dataset^11^ and Lewis Binford’s Hunter-Gatherer dataset^12^. Since the turn of the millennium, interest in cross-cultural databases has been stimulated by the possibilities offered by digital technologies. The *HRAF Collection* has been transformed into the online full-text *eHRAF World Cultures* database (https://ehrafworldcultures.yale.edu/), and many of the above-mentioned datasets have been integrated into the open-access database *D-PLACE*^2^ (https://d-place.org) along with other geographical, environmental, and linguistic data. The global linguistic data have also been extensively digitized, resulting in numerous databases, such as *Ethnologue*^13^ (https://www.ethnologue.com), *Glottolog*^14^ (http://glottolog.org), *WALS*^15^ (https://wals.info/), and *Grambank*^16^ (https://grambank.clld.org/). In addition, several specialized databases have been created in recent years, focusing on historical polities (*Seshat: Global History Databank*^17^; https://seshatdatabank.info/), religion (*Database of Religious History*^18^; https://religiondatabase.org/; *Pulotu*^19^; https://pulotu.com/), music (*Global Jukebox*^20^; https://theglobaljukebox.org/; *Natural History of Song Discography*^21^), and kinship terminologies (*Kinbank*^22^; http://www.kinbank.net/).

Here we present a lesser-known cross-cultural dataset created by the late Robert Carneiro and his colleagues in the 1960s and 1970s. Carneiro was a prominent anthropologist at the American Museum of Natural History (AMNH) who devoted his life to the study of political evolution and the evolution of complex societies. Through his *Scale Analysis* project^23–27^, he sought to resurrect interest in cultural evolution and rehabilitate the idea of unilinear cultural development. Like traditional evolutionists of the 19th century, Carneiro strove to uncover a general evolutionary sequence that human societies have consistently followed. But instead of defining cultural stages such as Morgan’s savagery, barbarism, and civilization^28^, he focused on the order in which societies tend to develop certain traits. He also pointed to the phenomenon of “differential evolution”, according to which development within one aspect of culture (e.g., economics) proceeds independently of evolution in other aspects (e.g., political organization). Carneiro’s approach to testing unilinear evolution was empirical, systematic, and comprehensive. The project’s name was derived from the scale analysis method, adopted from sociology^29,30^, which Carneiro used to demonstrate the existence of a regular scaling pattern in cultural traits. For this purpose, he collected ethnographic data on the presence or absence of hundreds of cultural traits in more than a hundred societies worldwide. He focused primarily on traits indicating a higher degree of cultural complexity that tend to accumulate and persist in society once developed. The trait list, however, was thematically very broad, ranging from surplus food production and class stratification, to judicial processes, temples, stone architecture, taxation, military conscription, metallurgy, and calendrical systems. The scale analysis of these synchronic cultural data, supplemented by a historical case study from Anglo-Saxon England^26^ showed the existence of certain cross-cultural regularities, based on which Carneiro inferred diachronic evolutionary processes and a “historical” sequence of cultural development^24,25,27^. For example, he concluded that religious practitioners generally preceded craft specialization, which preceded sumptuary laws. In addition, the resulting trait list was also proposed to serve as a measure of cultural complexity^31^, called *Index of Cultural Accumulation*, similar to the scales created by Naroll^32^ or Murdock and Provost^33^.

Given that Carneiro and his team created the dataset more than 50 years ago, it unfortunately but logically does not meet some recent standards for coding cultural data^1,34,35^. This applies especially to the principles of open science, such as transparency and documentation of research methodologies. Carneiro states that the main unit of analysis was “maximal socio-political unit”^36^, but apart from the name of the society, he rarely specifies its exact location or geographical boundaries. Nor does he usually mention the focal time to which the data were coded. Furthermore, the dataset lacks a detailed codebook according to which each variable was coded, information on how particular coding decisions were made, how uncertainty arising from ambiguity and contradictory statements was addressed, how variation within populations was treated, and how ethnographic biases were dealt with. Although academic references or coders’ notes are sometimes provided for data entries, this is unfortunately far from the norm. This is particularly problematic in the case of evidence of absence, where it is unclear whether a cultural trait was coded as absent because the sources explicitly describe its absence or because the coder merely inferred it. All of this makes it difficult to evaluate the reliability and validity of the dataset.

However, many of these limitations apply to other ethnography-based cross-cultural datasets as well^1^. Yet they are still considered valuable for cross-cultural and evolutionary research, and at least for the Ethnographic Atlas, a recent evaluation has confirmed the validity of its data^37^.

Carneiro’s dataset represents a unique and extensive set of cross-cultural data, coded independently of other major cross-cultural datasets, that has not been publicly available until now. Only a list of societies, cultural traits, and the main results of the analyses have been published^24,25,27^. The dataset itself was stored in the archives of the American Museum of Natural History, where we found it in 2022 and decided to digitize it. Our goal is to make the dataset accessible to the scientific community and, by transforming it into a machine-readable format, enable its reuse in future research.

Unfortunately, Carneiro passed away in 2020, so it was not possible to consult with him about the digitization project. His publications and notes indicate that the dataset was produced in several versions. We digitized the two that he primarily referred to: the 4th edition with 127 societies and 354 cultural traits, and the 6th edition with 74 societies and 618 traits. In addition to publishing them as separate datasets on Zenodo, we have also incorporated them into the D-PLACE database^2^, where they are accessible in a user-friendly interface and linked to other cultural, environmental and linguistic datasets. Researchers can use Carneiro’s datasets as a comparative and validation resource for other cross-cultural datasets, or as a starting point for collecting their own data and quantitatively testing cross-cultural hypotheses.

## Methods

### Original data collection

Details about Carneiro’s workflow of data collection and coding are unfortunately limited, based solely on his publications and notes^23–27,36^. Nevertheless, the basic concepts are clear. The units of the dataset are human societies, and the attributes are cultural traits.

Societies were defined as “maximal socio-political units”, meaning “they represented groups which, while in themselves continuously integrated, were not at the same time permanent sub-units of some larger socio-political unit”^36^. Consequently, societies vary in type and size, ranging from bands and villages to kingdoms and empires. While most societies are from the recent ethnographic (post)contact era, the dataset also includes several historical cases of early civilizations such as the Roman Empire and New Kingdom Egypt. Although cultural traits for each society were coded at the single time level^25^, the specific focal time is unfortunately only provided for historical cases and is missing for most societies.

The sample of societies was stratified to provide the broadest possible coverage of cultural complexity and geographical distribution^23^. A special effort was made to “include societies which in certain respects are unusual or anomalous, such as the Warao, with their unexpected priest-temple cult, the Kwakiutl, with their notable cultural elaboration despite the absence of agriculture, horse-riding hunters like the Blackfoot, Comanche, and Tehuelche, and pastoral nomads like the Lapps, Rwala, and Tuareg”^24^. The most recently published sample contained two lists of societies: one with 100 societies and 354 cultural traits, and the other with 56 societies and 618 traits^27^. However, Carneiro’s notes included an updated list of 72 societies with 618 cultural traits dated to September 14, 1970, and several other coded but unpublished societies. It seems that the unfinished aim was a sample of 200 societies.

Since the project focused on scale analysis, Carneiro and his colleagues were primarily interested in cultural traits expected to show cumulation and scaling. Specifically, the traits had to meet the following four criteria: “(1) Once developed or adopted by a society, the trait should be retained indefinitely, rather than being superseded at a later date. We therefore excluded such traits as cannibalism and head deformation, which arise at intermediate cultural levels but do not often survive into later stages. (2) The trait should not be universal, or virtually so, among human societies, since such a trait, even though it would scale, would not permit us to distinguish between one society and another. Thus, we did not include such traits as fire making, the incest taboo, and a belief in personal souls, all of extremely wide distribution. (3) If of limited distribution, the trait should not be found only among societies with relatively small cultural inventories. Thus, marriage classes were excluded, since they occur in Aboriginal Australia and virtually nowhere else. (4) The trait should not be sporadically distributed among societies of all cultural levels. Accordingly, we omitted traits like cremation and patrilocal residence, which are of irregular occurrence”^24^. Elsewhere, Carneiro notes that they selected for traits whose “presence indicates a greater degree of complexity than their absence”^23^ and that “[d]eciding whether a trait seemed likely to scale well was done based on a general knowledge of ethnography, history, and prehistory. No strict rule was applied”^27^.

The list of traits was revised several times. The 4th edition, which is most frequently mentioned in Carneiro’s publications, dates back to April 1963. It consists of 354 traits arranged into 14 categories, including Subsistence, Settlements, Architecture, Economics, Social Organization and Stratification, Political Organization, Law and Judicial Process, Warfare, Religion, Ceramics and Art, Tools, Utensils, and Textiles, Metalworking, Watercraft and Navigation, Special Knowledge and Practices. Later, the phrasing of some traits was refined and the list was considerably expanded, with the final 6th edition (dated to June 1965) containing 618 traits^27^. The 5th edition (dated to September 1964) also contains 618 traits and appears to have served as a preliminary version of the 6th edition. In fact, Carneiro’s 6th-edition list of societies includes some for which coding sheets exist only from the 5th edition, suggesting that the two editions were interchangeable in practice.

Cultural traits were coded in binary form, as *present*, marked with a plus sign ( + ), or *absent*, marked with a minus sign (−). Uncertainty regarding coding was occasionally marked with a question mark? or parentheses (). Binary coding of presence or absence was chosen for practical reasons because detailed quantitative information on the frequency of the studied traits is rarely found in the ethnographic and historical literature^27^.

Some data was coded by ethnographers specializing in the particular culture, including Carneiro himself, but most of the data was apparently collected by undergraduate students “by reading ethnographic monographs”^23–25^. For some societies, the name of the expert or coder is noted, but for many others, this information is unfortunately missing. Thus, it is unclear whether more than one coder was used for a particular culture, to what extent the data collected by students was reviewed by experts, and how ambiguities and disagreements about coding decisions were resolved. However, the coding sheets indicate that the codes were regularly checked and corrected, apparently by Carneiro. They were also occasionally updated, hence the final count of traits present differs from those previously published.

For most societies, a list of references or the name of the ethnographer is provided as the source of information. However, when it comes to citations or comments on particular coding decisions, the record is very uneven. In some cases, the documentation is detailed, while in others there is practically none.

### Datasets digitization

**Fig. 1:**
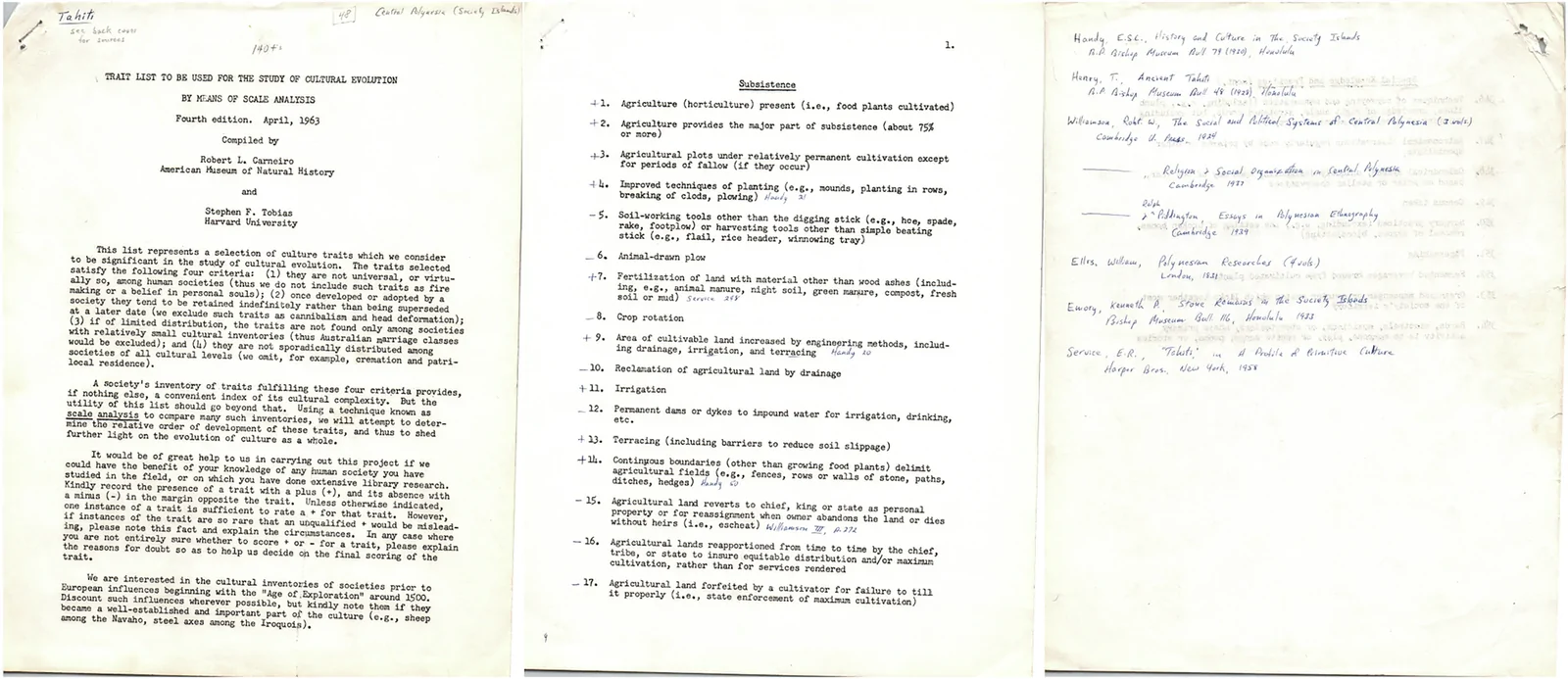
A sample of several pages of coding sheets from the 4th edition.

First, we scanned the original coding sheets, or trait lists, which are stored in the archives of the American Museum of Natural History (Fig. 1). Then, with the help of student assistants, we manually transcribed the data into Excel spreadsheets, which we converted to CLDF format^38^. CLDF is a package format for tabular data widely used in comparative linguistics. It is well-suited to encode categorical variables coded for societies which can be identified by the language spoken.

Of the six editions of the datasets, we focused only on the 4th and 6th. For the 4th edition, we digitized all 100 societies that Carneiro used for his published analysis^27^ and added another 27 societies for which we found complete or nearly complete coded data. We chose 10 or fewer missing data points as our cut-off criterion. For the 6th edition, we digitized 66 of the 72 societies included in Carneiro’s list from September 1970 (unpublished), to which we added another 8 societies with complete coded data (namely Zulu, Eauripik, Klallam, Xikrin, Seri, Agta, Chippewyan, and Kubu). We did not include six societies from the 1970 list (namely Ainu, Aleuts, Kaoka, Kipsigis, Maori and Marshallese) because their coding sheets could not be matched with the trait list. It was only a numerical list, with no trait descriptions, and had 617 items instead of 618. See Supplementary Information for a detailed list of societies.

The societies in the original dataset were identified by name, occasionally supplemented by a geographical area. In order to link the data with other cross-cultural databases, we added glottocodes^39^ and approximate geographical coordinates to each society, and, where possible, also eHRAF World Cultures ID. We paired Carneiro’s dataset with these databases based on the same society name and/or the same references used for data coding. Information on latitude and longitude was primarily obtained from the Ethnographic Atlas^8^ (via D-PLACE^2^), or from Glottolog^14^, or the choice of coordinates documented in the notes. To facilitate linking with D-PLACE, we have adopted their preferred English-language ethnonyms, but we also retained the original society names used by Carneiro. We only recorded focal times that were in the original coding sheets and did not attempt to complete them.

The names/descriptions of cultural traits for the 4th edition were taken from coding sheets, while those for the 6th edition were taken from Carneiro’s paper^27^, which we assume was the most up-to-date. Apart from these brief descriptions, we did not find any more detailed definitions of individual traits or instructions on how to code them. For practical reasons (e.g., better display on the D-PLACE website), we shortened the names of some traits, leaving the original names only as descriptions. Since there is a partial overlap between the trait lists from the 4th and 6th editions, we added two types of notes to the traits in the 6th edition: “Note, identical to” in the case of traits whose descriptions were exactly the same as those in the 4th edition, and “Note, similar to” in the case of traits that were basically the same as those in the 4th edition but their descriptions were slightly different.

We manually transcribed the original values in the form of pluses and minuses into numerical ones and zeros. We did not record the notes on the uncertainties of the coding decision (e.g., question marks or pluses in parentheses) in any special way. If they were present, we included them in the notes along with the other original comments. To minimize transcription errors, each generated spreadsheet was double-checked. If any original values and comments were illegible, this was always noted. References used in the original creation of the dataset were also recorded and additionally transcribed into BibTeX format.

We provide the transcribed data in two forms: (1) “raw” Excel and CSV spreadsheets, and (2) CLDF format. CLDF is particularly suited for this data because the D-PLACE database is aggregated from individual datasets in CLDF format (e.g. the Ethnographic Atlas). So by providing the Carneiro data in a compatible format, we make sure it can be easily integrated into D-PLACE.

The datasets are converted to CLDF *StructureDatasets* - a format typically used for typological survey data. Societies are listed in a dataset’s *LanguageTable*. This choice makes sense because in CLDF datasets languages are the objects of study, and societies in cross-cultural datasets share many metadata properties of languages, such as an approximate geographic location or alternative names. Traits are listed in the *ParameterTable* and categories, i.e. the range of possible values for a trait, are listed in the *CodeTable*. We use the *cldfbench* tool^40^ to implement the conversion from raw tables to CLDF in a reproducible way. This guarantees that the CLDF data can be recreated when errors are fixed in the raw data or to integrate changes in external data, such as new Glottolog versions.

## Data Records

Both datasets are archived with and published under a CC-BY 4.0 license on Zenodo^41,42^.

The files for each dataset are arranged in the typical structure of cldfbench-curated datasets:

- A directory ‘raw’ holds the data “as digitized”, i.e. as Excel sheets filled in during transcription.
- A directory ‘cldf’ holds the files of the CLDF dataset. These files can be recreated from the raw data by running the ‘cldfbench makecldf’ command, which in turn will execute the custom conversion code in ‘cldfbench_carneiro?.py’.
- Various documents formatted in markdown provide additional information typically expected from data or code repositories such as a ‘README.md’ or ‘LICENSE’.

The main data intended for re-use is the CLDF dataset, which we will describe in more detail in the remainder of this section. The CLDF dataset consists of CSV-files and metadata following the W3C recommendation CSVW (CSV on the Web). The machine-readable metadata in ‘StructureDataset-metadata.json’ is also used by *cldfbench* to create a human-readable, markdown-formatted README.

Data files are formatted as UTF-8 encoded CSV files and thus can easily be accessed from any computing platform. Thus, while analysis workflows may make use of the machine-readability of the metadata (e.g. to infer data types), the same knowledge may be gleaned from the human-readable metadata and added “manually” to analyses.

‘societies.csv’ includes the necessary information about the societies in the datasets: ID, Name (both original and modified), Glottocode, Latitude, Longitude, Region and Main Focal Year of the society. It also contains additional comments and eHRAF World Cultures name and ID to facilitate linking to this database.

‘variables.csv’ stores information about the cultural traits, including their ID, Name, Description, Category and Type. Identifiers in ‘variables.csv’ are used as foreign keys in ‘codes.csv’ to link possible values for a trait to the trait description. ‘codes.csv’ includes a list of all code values for all cultural traits, with columns for the code ID, trait ID (the foreign key), Name/Description (i.e. absent or present) and Value (i.e., 0 or 1). The individual coded data-points are stored in ‘data.csv’, with columns for the entry ID, the society ID (a foreign key into ‘societies.csv’), a trait ID (a foreign key into ‘variables.csv’), value, a code ID (a foreign key into ‘codes.csv’), additional comments, the source, and the year to which the data relate. The ‘sources.bib’ file contains all the sources that were used as references for the dataset in BibTeX-format. The ‘requirements.txt’ and ‘README.md’ files round off the folder for reproducibility of the CLDF conversion. Entries in this BibTeX file are referenced in the source column of ‘data.csv’.

The original raw data is available as Excel-files and CSV-files within a directory ‘?TH_EDITION/societies’ in the ‘raw’-folder. The ‘raw’ folder also holds additional metadata which informed the conversion to CLDF such as metadata about societies and sources.

## Data Overview

**Fig. 2:**
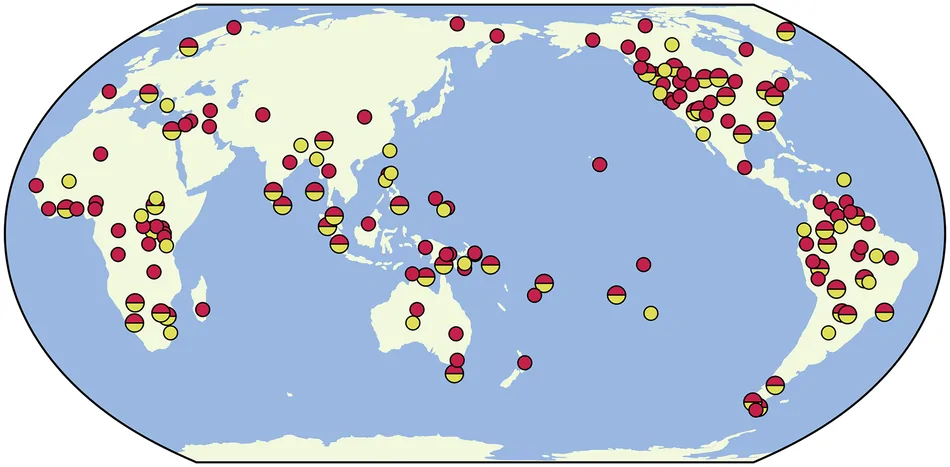
Distribution of societies in the 4th (red) and 6th (yellow) editions of Carneiro’s dataset.

The datasets together include 152 societies, of which 49 are in both editions, 78 are only in the 4th edition, and 25 are only in the 6th edition (Fig. 2; Supplementary Information). The 4th edition contains binary data on the presence of 354 cultural traits, while the 6th edition contains 618 traits. In both datasets, the traits are divided into the same 14 categories (Table 1). 162 variables are identical in both datasets, and 164 are similar (i.e., the same cultural trait but with slightly different definition), meaning that 28 variables are unique in the 4th edition, while this number is 292 for the 6th edition.

**Table 1:** Number of traits in each category in each dataset.

Category	4th edition	6th edition
Subsistence	27	32
Settlements	29	38
Architecture	27	35
Economics	40	111
Social Organization and Stratification	19	42
Political Organization	51	90
Law and Judicial Process	20	43
Warfare	31	57
Religion	32	54
Ceramics and Art	16	17
Tools, Utensils, and Textiles	13	22
Metalworking	22	25
Watercraft and Navigation	9	17
Special Knowledge and Practices	18	35

## Technical Validation

To minimize errors that may have occurred during transcription, each generated spreadsheet was double-checked. For most societies (100 from the 4th edition and 72 from the 6th edition), we further checked the total number of cultural traits reported as present. We note that in several cases the values differ (see Supplementary Information). After careful checking, we state that these differences are not caused by transcription errors, but by discrepancies between the published information and the values in the coding sheets. In such cases, we have given priority to the information in the coding sheets, as we assume that they represent the most recent version of the record.

Thanks to the CLDF standard, basic validation of the data, including ensuring relational integrity, could be checked by running the validation tool provided by the *pycldf* package^43^. Additional validation of the metadata is implemented in the ‘check’ command of the *pydplace* package, which we could run because the data model of our datasets is compatible with D-PLACE.

## Usage Notes

In addition to the Zenodo repositories, the datasets are accessible in a user-friendly interface at the D-PLACE website (https://d-place.org/contributions/dplace-dataset-carneiro4 and https://d-place.org/contributions/dplace-dataset-carneiro6). There, users can find basic summary statistics, search for and explore specific societies and cultural traits, and visualize their distribution on a world map, as well as on selected linguistic phylogenies. Compatibility with the other datasets aggregated in D-PLACE also means that the “recipes” for using D-PLACE data collected at https://github.com/D-PLACE/dplace-cookbook are largely applicable to our datasets.

For instructions on how to extract CLDF data for selected variables, see this how-to guide https://github.com/D-PLACE/dplace-cookbook/tree/master/recipes/extracting_variables.

Since these are legacy datasets with limited documentation of the original coding procedures, we recommend users to recode a sample of data to understand how it was coded and to assess its quality. We also recommend interpreting the value “absent” more as “absent or no evidence.”

Those interested in Carneiro’s original coding sheets can download them from Zenodo repository^44^. The folder contains not only the scans we used to digitize the datasets, but also many other notes on the Scale Analysis project. However, due to time constraints, not everything has been scanned, and the full archive is available in physical form at the American Museum of Natural History (*Carneiro’s Scale Analysis notes*, catalogue number C376, boxes 1 to 5).

Finally, we note that the digitization process has preserved the records in their original form without modification. However, users should bear in mind that these cultural data were collected and coded according to anthropological practices of the mid-twentieth century, and may not align with contemporary CARE principles for Indigenous Data Governance^45^.

## Supplementary information

Supplementary Information

## Data Availability

Both datasets are archived with and published on Zenodo (4th edition https://doi.org/10.5281/zenodo.17607789; 6th edition https://doi.org/10.5281/zenodo.17607723). Scans of Carneiro’s original coding sheets are available at https://doi.org/10.5281/zenodo.18325159.

## Code availability

The code to convert the digitized coding sheets to CLDF is available within the datasets (see https://github.com/D-PLACE/dplace-dataset-carneiro4/blob/main/cldfbench_carneiro4.py). It uses the Python package pydplace, available from the Python Package Index at https://pypi.org/project/pydplace.

## References

[1] Watts, J. *et al*. Building Quantitative Cross-Cultural Databases From Ethnographic Records: Promise, Problems and Principles. *Cross-Cultural Research* **56**, 62–94 10.1177/10693971211065720 (2022).

[2] Kirby, K. R. *et al*. D-PLACE: A Global Database of Cultural, Linguistic and Environmental Diversity. *PLOS ONE* **11**, e0158391 10.1371/journal.pone.0158391 (2016). PMC4938595 27391016

[3] Gray, R. D. & Watts, J. Cultural macroevolution matters. *Proceedings of the National Academy of Sciences* **114**, 7846–7852 (2017). 10.1073/pnas.1620746114 PMC5544274 28739960

[4] Fogarty, L., Kandler, A., Creanza, N. & Feldman, M. W. Half a century of quantitative cultural evolution. *Proceedings of the National Academy of Sciences* **121**, e2418106121 (2024). 10.1073/pnas.2418106121 PMC11621465 39556730

[5] Kroeber, A. L. Culture Element Distributions: XI Tribes Surveyed. *Anthropological Records* **1**, 435–440 (1939).

[6] Murdock, G. P. Feasibility and implementation of comparative community research: With special reference to the Human Relations Area Files. *American sociological review* **15**, 713–720 (1950).

[7] Murdock, G. P. *et al*. *Outline of Cultural Materials. 6th revised edition with modifications*. (Human Relations Area Files, 2008).

[8] Murdock, G. P. Ethnographic atlas: a summary. *Ethnology* **6**, 109–236 (1967).

[9] Murdock, G. P. & White, D. R. Standard Cross-Cultural Sample. *Ethnology* **8**, 329–369 10.2307/3772907 (1969).

[10] Textor, R. B. *A Cross-Cultural Summary*. (HRAF Press, 1967).

[11] Jorgensen, J. G. *Western Indians: Comparative environments, languages, and cultures of 172 Western American Indian tribes*. (WH Freeman, 1980).

[12] Binford, L. R. *Constructing frames of reference: an analytical method for archaeological theory building using ethnographic and environmental data sets*. (University of California Press, 2001).

[13] Eberhard, D. M., Simons, G. F. & Robinson, A. J. *Ethnologue: Languages of the World. Twenty-ninth edition*, https://www.ethnologue.com/ (2026).

[14] Hammarström, H., Forkel, R., Haspelmath, M. & Bank, S. *Glottolog 5.3* https://glottolog.org/ (2026).

[15] Dryer, M. S. & Haspelmath, M. *The World Atlas of Language Structures Online*, http://wals.info (2013).

[16] Skirgård, H. *et al*. Grambank reveals the importance of genealogical constraints on linguistic diversity and highlights the impact of language loss. *Science Advances* **9**, eadg6175 10.1126/sciadv.adg6175 (2023). PMC10115409 37075104

[17] Turchin, P. *et al*. Seshat: The Global History Databank. *Cliodynamics: The Journal of Quantitative History and Cultural Evolution* **6**, 77–107 (2015).

[18] Slingerland, E. & Sullivan, B. Durkheim with Data: The Database of Religious History. *Journal of the American Academy of Religion* **85**, 312–347 10.1093/jaarel/lfw012 (2017).

[19] Watts, J. *et al*. Pulotu: Database of Austronesian Supernatural Beliefs and Practices. *PLOS ONE* **10**, e0136783 10.1371/journal.pone.0136783 (2015). PMC4580586 26398231

[20] Wood, A. L. *et al*. The Global Jukebox: A public database of performing arts and culture. *PloS one* **17**, e0275469 (2022). 10.1371/journal.pone.0275469 PMC9629617 36322519

[21] Bertolo, M. *et al*. The Expanded Natural History of Song Discography, a global corpus of vocal music. *Open Mind* **9**, 844–863 (2025). 10.1162/opmi.a.4 PMC12283151 40697896

[22] Passmore, S. *et al*. Kinbank: A global database of kinship terminology. *Plos one* **18**, e0283218 (2023). 10.1371/journal.pone.0283218 PMC10208500 37224178

[23] Carneiro, R. L. Scale analysis as an instrument for the study of cultural evolution. *Southwestern Journal of Anthropology* **18**, 149–169 (1962).

[24] Carneiro, R. L. & Tobias, S. F. The application of scale analysis to the study of cultural evolution. *Transactions of the New York Academy of Sciences* **26**, 196–207 (1963).

[25] Carneiro, R. L. Ascertaining, testing, and interpreting sequences of cultural development. *Southwestern Journal of Anthropology* **24**, 354–374 (1968).

[26] Carneiro, R. L. The Measurement of Cultural Evolution in the Ancient. Near East and in Anglo-Saxon England. *Transactions of the New York Academy of Sciences* **31**, 1013–1023 (1969).

[27] Carneiro, R. L. Scale analysis, evolutionary sequences, and the rating of cultures in *A Handbook of Method in Cultural Anthropology* (eds R Naroll & R Cohen) 834-871 (1970).

[28] Morgan, L. H. *Ancient society: researches in the lines of human progress from savagery through barbarism to civilization*. (H. Holt and Company, 1877).

[29] Guttman, L. A basis for scaling qualitative data. *American sociological review* **9**, 139–150 (1944).

[30] Guttman, L. The Basis for Scalogram Analysis in *Studies in Social Psychology in World War II. VOLUME IV: Measurement and Prediction* (eds S A. Stouffer et al.) 60-90 (Princeton University Press, 1950).

[31] Chick, G. Cultural Complexity: The Concept and Its Measurement. *Cross-Cultural Research* **31**, 275–307 10.1177/106939719703100401 (1997).

[32] Naroll, R. A preliminary index of social development. *American anthropologist* **58**, 687–715 (1956).

[33] Murdock, G. P. & Provost, C. Measurement of Cultural Complexity. *Ethnology* **12**, 379–392 10.2307/3773367 (1973).

[34] Ember, C. R. & Ember, M. *Cross-cultural Research Methods*. (Altamira Press, 2009).

[35] Slingerland, E. *et al*. Coding culture: challenges and recommendations for comparative cultural databases. *Evolutionary Human Sciences* **2**, e29 10.1017/ehs.2020.30 (2020). PMC10427479 37588354

[36] Carneiro, R. L. On the relationship between size of population and complexity of social organization. *Southwestern Journal of Anthropology* **23**, 234–243 (1967).

[37] Bahrami-Rad, D., Becker, A. & Henrich, J. Tabulated nonsense? Testing the validity of the Ethnographic Atlas. *Economics Letters* **204**, 109880 (2021).

[38] Forkel, R. *et al*. Cross-Linguistic Data Formats, advancing data sharing and re-use in comparative linguistics. *Scientific data* **5**, 180205 (2018). 10.1038/sdata.2018.205 PMC6190742 30325347

[39] Forkel, R. & Hammarström, H. Glottocodes: Identifiers linking families, languages and dialects to comprehensive reference information. *Semantic Web* **13**, 917–924 (2022).

[40] Forkel, R. & List, J.-M. CLDFBench: Give your cross-linguistic data a lift in *Proceedings of the Twelfth Language Resources and Evaluation Conference*. (eds N Calzolari *et al.*) 6995-7002 (European Language Resources Association, 2020).

[41] Carneiro, R. L. D-PLACE dataset derived from Robert L. Carneiro’s Dataset (4th Edition) (v1.0.1). *Zenodo*10.5281/zenodo.17607789 (2025).

[42] Carneiro, R. L. D-PLACE dataset derived from Robert L. Carneiro’s Dataset (6th edition) (v1.0.1). *Zenodo*10.5281/zenodo.17607723 (2025).

[43] Forkel, R., Bank, S., Greenhill, S. J., Rzymski, C. & Kaiping, G. A. cldf/pycldf: pycldf (v1.6.4). *Zenodo*10.5281/zenodo.3355430 (2019).

[44] Carneiro, R. L. Scans of Robert L. Carneiro’s Scale Analysis notes. *Zenodo*10.5281/zenodo.18325159 (2026).

[45] Carroll, S. R. *et al*. The CARE Principles for Indigenous Data Governance. *Data Science Journal*10.5334/dsj-2020-043 (2020).

