# Supplementary material for: Carneiro’s global dataset of cultural traits in ethnographic and historical societies: a digital edition
Source: Sci Data. 2026 Oct 2;13:1378. doi: 10.1038/s41597-026-08212-0 (PMC13633262; doi:10.1038/s41597-026-08212-0)
Supplement: Supplementary file 1 [file 41597_2026_8212_MOESM1_ESM.pdf]

Supplementary Information

List of societies included in Carneiro's datasets. The numbers before the parentheses indicate the order in Carneiro's lists. The numbers in parentheses indicate the number of cultural traits that the society has; values that differ from those stated in the lists are marked in red. Six societies from the 6th edition list are not included in the digitized dataset (marked as *NA*) because their coding sheets could not be matched with the trait list. The ID refers to Carneiro's original coding sheets (scans of which are available at https://doi.org/10.5281/zenodo.18325159), as well as the box and folder in which the physical documents are stored in the AMNH archive (catalogue number C376).

Society	ID	List of 100 (4th ed.)[1]	List of 72 (6th ed.)[2]	Dataset (4th ed.)	Dataset (6th ed.)
New Kingdom Egypt (1350 BC)	b2f30	001 (329+)	003 (538+)	YES (329+)	YES (538+)
Roman Empire (100 AD)	b4f25	002 (323+)	001 (572+)	YES (323+)	YES (571+)
Ancient India (250 BC)	b2f52	-	002 (566+)	-	YES (566+)
Periclean Athens	b1f30	-	004 (486+)	-	YES (486+)
Assyrian Empire (650 BC)	b1f28	003 (311+)	-	YES (311+)	-
Aztecs	b1f32	004 (303+)	-	YES (302+)	-
China (Han Dynasty)	b2f43	005 (299+)	-	YES (299+)	-
Incas	b2f51	006 (288+)	-	YES (287+)	-
Kingdom of León	b3f34	007 (271+)	-	YES (271+)	-
Vikings	b5f20	008 (225+)	005 (344+)	YES (225+)	YES (344+)
Dahomey	b2f23	009 (202+)	-	YES (202+)	-
Ashanti	b1f27	010 (198+)	006 (315+)	YES (198+)	YES (315+)
Baganda	b1f33	011 (181+)	008 (253+)	YES (181+)	YES (253+)
Marquesans	b3f43	012 (142+)	-	YES (142+)	-
Tahitians	b4f39	013 (140+)	007 (254+)	YES (140+)	YES (254+)
Dogon	b2f25	-	009 (208+)	-	YES (208+)
Azande	b1f31	-	010 (184+)	-	YES (184+)
Hawaiians	b2f45	014 (127+)	-	YES (127+)	-
Mano	b3f40	015 (126+)	-	YES (126+)	-
Bavenda	b1f38	016 (124+)	012 (170+)	YES (124+)	YES (171+)
Batak	b1f37	017 (119+)	013 (160+)	YES (119+)	YES (162+)
Schilluk	b4f32	-	014 (138+)	-	YES (139+)
Mru	b4f7	-	015 (137+)	-	YES (137+)
Mangareva	b3f38	-	016 (134+)	-	YES (134+)
Fijians	b2f35	018 (114+)	-	YES (114+)	-

Bemba	b1f39	019 (100+)	-	YES (100+)	-
Suku	b4f37	020 (99+)	-	YES (99+)	-
Tanala	b4f42	021 (94+)	-	YES (94+)	-
Maori	b3f42	022 (92+)	021 (120+)	YES (91+)	*NA*
Pokot	b4f24	023 (86+)	-	YES (86+)	-
Acoma	b1f11	024 (82+)	-	YES (82+)	-
Rwala	b4f26	025 (79+)	-	YES (79+)	-
Creek	b2f19	026 (77+)	017 (131+)	YES (76+)	YES (132+)
Tuareg (Ahaggar)	b5f14	027 (76+)	-	YES (76+)	-
Kayan	b3f14	028 (72+)	-	YES (72+)	-
Marshallese	b3f44	-	019 (122+)	-	*NA*
Futunans	b2f37	029 (71+)	020 (120+)	YES (71+)	YES (120+)
Kofyar	b3f20	030 (70+)	-	YES (70+)	-
Thonga	b5f3	031 (68+)	011 (171+)	YES (68+)	YES (171+)
Masai	b3f45	-	022 (117+)	-	YES (118+)
Iroquois	b3f1	032 (64+)	023 (94+)	YES (64+)	YES (94+)
Bontoc	b2f2	033 (60+)	-	YES (60+)	-
Boloki	b2f1	033 (60+)	-	YES (60+)	-
Kwakiutl	b3f28	035 (57+)	-	YES (57+)	-
Molima	b4f5	036 (56+)	-	YES (56+)	-
Elgeyo	b2f31	037 (55+)	-	YES (55+)	-
Chiga	b2f14	038 (52+)	-	YES (52+)	-
Karen	b3f12	039 (51+)	-	YES (51+)	-
Lango	b3f30	040 (50+)	-	YES (50+)	-
Ao Naga	b1f24	041 (49+)	018 (125+)	YES (49+)	YES (126+)
Kipsigis	b3f17	-	024 (90+)	YES (49+)	*NA*
Nootka	b4f14	-	025 (78+)	YES (54+)	YES (79+)
Mandan	b3f37	042 (47+)	026 (77+)	YES (47+)	YES (78+)
Callinago	b2f6	-	027 (75+)	-	YES (75+)
Trobriands	b5f12	-	028 (74+)	-	YES (75+)
Omaha	b4f17	043 (45+)	040 (51+)	YES (45+)	YES (51+)
Ifaluk	b2f50	043 (45+)	-	YES (45+)	-
Nama Hottentot	b2f48	045 (44+)	031 (62+)	YES (44+)	YES (63+)
Menomini	b4f2	046 (43+)	-	YES (43+)	-
Navajo	b4f11	-	032 (61+)	YES (50+)	YES (62+)

Aleut	b1f18	-	033 (60+)	-	*NA*
Manobo	b3f41	047 (41+)	034 (59+)	YES (41+)	YES (60+)
Atayal	b1f29	-	035 (59+)	-	YES (58+)
Tupinambá	b5f16	048 (40+)	042 (50+)	YES (40+)	YES (50+)
Siuai	b4f35	049 (39+)	-	YES (39+)	-
Cheyenne	b2f12	049 (39+)	-	YES (39+)	-
Flathead	b2f36	051 (38+)	-	YES (38+)	-
Kiwai	b3f18	052 (37+)	029 (71+)	YES (37+)	YES (72+)
Gururumba	b2f42	053 (36+)	-	YES (36+)	-
Toda	b5f10	053 (36+)	036 (58+)	YES (36+)	YES (58+)
Klallam	b3f19	055 (34+)	-	YES (34+)	YES (60+)
Mala	b3f36	055 (34+)	030 (66+)	YES (34+)	YES (65+)
Reindeer Lapps	b3f31	057 (33+)	-	YES (33+)	-
Guaraní	b2f41	058 (31+)	048 (36+)	YES (31+)	YES (36+)
Yukaghir	b5f35	059 (30+)	-	YES (30+)	-
Nuer	b4f15	060 (28+)	037 (57+)	YES (28+)	YES (57+)
Tolowa	b5f11	-	038 (55+)	-	YES (57+)
Ainu	b1f16	-	039 (55+)	-	*NA*
Delaware	b2f24	-	041 (50+)	YES (30+)	YES (50+)
Cayapa	b2f10	-	043 (44+)	-	YES (44+)
Kaoka	b3f9	-	044 (43+)	-	*NA*
Kutenai	b3f27	-	045 (41+)	-	YES (41+)
Abipones	b1f14	-	047 (38+)	-	YES (38+)
Tanaina	b4f45	061 (27+)	-	YES (27+)	-
Tukuna	b5f15	062 (25+)	050 (31+)	YES (25+)	YES (31+)
Comanche	b2f17	062 (25+)	-	YES (25+)	-
Tenino	b5f2	064 (24+)	-	YES (24+)	-
Blackfoot	b1f41	065 (23+)	049 (31+)	YES (23+)	YES (31+)
Havasupai	b2f44	065 (23+)	053 (28+)	YES (23+)	YES (28+)
Zambales Negrito	b5f37	-	054 (24+)	-	YES (24+)
Yanomamö	b5f30	-	055 (23+)	-	YES (23+)
Shavante	b2f11	-	056 (22+)	-	YES (22+)
Canela	b2f8	065 (23+)	-	YES (23+)	-
Warao	b5f22	065 (23+)	-	YES (23+)	-
Yupa	b5f36	069 (22+)	-	YES (22+)	-

Koryak	b3f22	069 (22+)	-	YES (22+)	-
Jívaro	b3f5	069 (22+)	-	YES (22+)	-
Kamar	b3f8	072 (21+)	-	YES (21+)	-
Kuikuru	b3f24	073 (19+)	051 (28+)	YES (19+)	YES (28+)
Tehuelche	b5f1	074 (18+)	046 (40+)	YES (18+)	YES (40+)
Mundurucú	b4f8	074 (18+)	-	YES (18+)	-
Gros Ventres	b2f40	074 (18+)	-	YES (18+)	-
Barama River Caribs	b1f36	074 (18+)	057 (21+)	YES (18+)	YES (21+)
Campa (Asháninka)	b2f7	078 (17+)	-	YES (17+)	-
Yaruro	b5f32	079 (16+)	-	YES (16+)	-
Cubeo	b2f21	080 (14+)	052 (28+)	YES (14+)	YES (28+)
Northern Maidu	b3f35	081 (13+)	-	YES (13+)	-
Amahuaca	b1f20	082 (11+)	063 (15+)	YES (11+)	YES (15+)
Copper Eskimo	b2f18	083 (10+)	-	YES (10+)	-
Chippewa	b2f16	083 (10+)	058 (19+)	YES (10+)	YES (19+)
Andamanese	b1f22	083 (10+)	064 (15+)	YES (10+)	YES (15+)
Ammassalik	b1f21	083 (10+)	062 (16+)	YES (10+)	YES (16+)
Lengua	b3f33	087 (9+)	059 (19+)	YES (9+)	YES (20+)
Karankawa	b3f11	-	060 (18+)	YES (11+)	YES (18+)
Sirionó	b4f34	088 (7+)	069 (9+)	YES (7+)	YES (9+)
Vedda	b5f19	088 (7+)	065 (14+)	YES (7+)	YES (14+)
Pitjantjatjara	b4f23	-	066 (14+)	-	YES (14+)
Kaska	b3f13	088 (7+)	-	YES (7+)	-
Naskapi	b4f10	091 (6+)	-	YES (6+)	-
Bushmen (NW groups)	b2f5	092 (5+)	067 (13+)	YES (5+)	YES (13+)
Yahgan	b5f28	092 (5+)	-	YES (5+)	-
Walbiri	b5f21	092 (5+)	-	YES (5+)	-
Murngin	b4f9	092 (5+)	061 (17+)	YES (5+)	YES (18+)
Washo	b5f23	096 (4+)	-	YES (4+)	-
Semang	b4f29	097 (3+)	068 (12+)	YES (3+)	YES (12+)
Kurnai	b3f25	097 (3+)	-	YES (3+)	-
BaMbuti Pygmies	b1f35	097 (3+)	-	YES (3+)	-
Tasmanians	b4f44	100 (0+)	071 (5+)	YES (0+)	YES (5+)
Ona	b4f18	-	070 (7+)	YES (5+)	YES (7+)
Alacaluf	b1f17	-	072 (3+)	YES (2+)	YES (3+)

Zulu	b5f38	-	-	-	YES (180+)
Eauripik	b2f29	-	-	-	YES (76+)
Xikrin	b5f26	-	-	-	YES (42+)
Seri	b4f31	-	-	-	YES (33+)
Agta	b1f15	-	-	-	YES (23+)
Chipewyan	b2f15	-	-	-	YES (19+)
Kubu	b3f23	-	-	YES (3+)	YES (8+)
Sumerians	b4f38	-	-	YES (257+)	-
Wolof	b5f25	-	-	YES (147+)	-
Hazaras	b2f46	-	-	YES (103+)	-
Tiv	b5f7	-	-	YES (72+)	-
Jericho (Pre-Pottery Neolithic)	b3f04	-	-	YES (57+)	-
Kapauku	b3f10	-	-	YES (45+)	-
Kakoli	b3f7	-	-	YES (40+)	-
Ulithi	b5f17	-	-	YES (39+)	-
Crow	b2f20	-	-	YES (38+)	-
Kurtatchi	b3f26	-	-	YES (25+)	-
Penobscot	b4f22	-	-	YES (25+)	-
Akawaio	b1f13	-	-	YES (21+)	-
Waiyana	b1f13	-	-	YES (20+)	-
Yabarana	b5f27	-	-	YES (13+)	-
Maué	b3f46	-	-	YES (11+)	-
Shoshone	b4f33	-	-	YES (8+)	-
Tiwi	b5f8	-	-	YES (6+)	-
Euahlayi	b2f33	-	-	YES (4+)	-
Sekani	b4f28	-	-	YES (4+)	-

[1]Based on Carneiro (1970: Table 2 in *A Handbook of Method in Cultural Anthropology*).
[2]Based on a list in Carneiro's archive, dated September 14, 1970.
